# Prevalence, Epidemiological Characteristics, and Pharmacotherapy of Coronary Artery Disease among Patients with Atrial Fibrillation: Data from Jo-Fib Study

**DOI:** 10.3390/medicina57060605

**Published:** 2021-06-11

**Authors:** Hanna K. Al-Makhamreh, Mohammed Q. Al-Sabbagh, Ala’ E. Shaban, Abdelrahman F. Obiedat, Ayman J. Hammoudeh

**Affiliations:** 1 Division of Cardiology, Department of Internal Medicine, Jordan University Hospital, University of Jordan School of Medicine, Amman 11942, Jordan; HMAKHAMREH@hotmail.com; 2 University of Jordan School of Medicine, Amman 11942, Jordan; a.shabban@hotmail.com (A.E.S.); aobiedat6@gmail.com (A.F.O.); 3 Department of neurology, University of Kansas Medical Center, Kansas City, KS 66160, USA; 4 Department of Cardiology, Istishari Hospital, Amman 11184, Jordan; hammoudeh_ayman@yahoo.com

**Keywords:** atrial fibrillation, coronary artery disease, Jordan, bleeding risk, thromboembolism

## Abstract

*Background and Objectives:* Patients with AF are at increased risk for Coronary Artery Disease (CAD) owing to their shared etiologies and risk factors. This study aimed to assess the prevalence, cardiovascular risk factors, and used medications of CAD in AF patients. *Materials and Methods:* This retrospective, case-control study utilized data from the Jordanian Atrial Fibrillation (Jo-Fib) registry. Investigators collected clinical features, history of co-existing comorbidities, CHA2DS2-VASc, and HAS BLED scores for all AF patients aged >18 visiting 19 hospitals and 30 outpatient cardiology clinics. A multivariable binary logistic regression was used to asses for factors associated with higher odds of having CAD. *Results:* Out of 2000 patients with AF, 227 (11.35%) had CAD. Compared to the rest of the sample, those with CAD had significantly higher prevalence of hypertension (82.38%; *p* < 0.01), hypercholesterolemia (66.52%, *p* < 0.01), diabetes (56.83%, *p* < 0.01), and smoking (18.06%, *p* = 0.04). Patients with AF and CAD had higher use of anticoagulants/antiplatelet agents combination (*p* < 0.01) compared to the rest of the sample. Females had lower CAD risk than males (OR = 0.35, 95% CI: 0.24–0.50). AF Patients with dyslipidemia (OR = 2.5, 95% CI: 1.8–3.4), smoking (OR = 1.7, 95% CI: 1.1–2.6), higher CHA2DS2-VASc score (OR = 1.5, 95% CI: 1.4–1.7), and asymptomatic AF (OR = 1.9, 95% CI: 1.3–2.6) had higher risk for CAD. *Conclusions:* Owing to the increased prevalence of CAD in patients with AF, better control of cardiac risk factors is recommended for this special group. Future studies should investigate such interesting relationships to stratify CAD risk in AF patients. We believe that this study adds valuable information regarding the prevalence, epidemiological characteristics, and pharmacotherapy of CAD in patients with AF.

## 1. Introduction

Atrial fibrillation (AF) is the most commonly diagnosed cardiac arrhythmia worldwide [1]. The risk of developing AF in the general population is around 2%, and it increases with age to the extent that it is found in 14% of those who are above 80 [2]. The risk of developing AF is especially higher in European ancestry, higher body weight, hypertension (HTN), diabetes mellitus (DM), and ischemic heart disease (IHD) [3,4]. AF is associated with worse mortality and morbidity outcomes worldwide; in a nationwide, population-based study, all-cause mortality in AF patients was 4 times more than that in the general population [5]. Moreover, in patients with non-valvular AF, the annual risk for arterial thromboembolism, including stroke, is 5%; this probability is even higher in patients older than 75 years and those with a previous history of stroke or transient ischemic attack (TIA) [5]. 

The prevalence of coronary artery disease (CAD) also increases with age, as it shares the common vascular risk factors of smoking, HTN, DM, and dyslipidemia with AF [6]. Patients with AF have higher prevalence of comorbid CAD in comparison to the general population; it is estimated that around 17% to 46.5% of patients with AF have a concurrent coronary artery disease (CAD) [7]. Myocardial infarctions (MI) might also predispose to AF or complicate the clinical course of preexisting AF [8]. It has been reported that one in ten patients presenting with MI had a previous history of AF [9]. Additionally, 6% to 21% of patients with MI will develop AF [10]. Therefore, the development of AF is a poor prognostic factor post-MI both in the short and the long term [9]. On the other hand, there is increasing evidence that AF might predispose to CAD [11]. AF is associated with systemic signs of inflammation that might lead to a pro-thrombotic status that predisposes to CAD and ultimately myocardial infarction (MI)[12].

Coexisting CAD and AF (CAD-AF) is a challenging situation for clinicians to manage, especially in the case of acute coronary syndrome (ACS) due to the complexity of the used anticoagulants and antiplatelet agents, with the subsequent excessive chance of bleeding [6]. Therefore, there is a growing need for a better understanding of the general characteristics and risk factors of patients with CAD-AF.

In this study, we aimed to identify the prevalence of coronary artery disease (CAD) in AF patients in Jordan. In addition, we compared the clinical risk factors, treatment, and outcomes of CAD-AF patients to the rest of AF patients. 

## 2. Methodology

### 2.1. Study Design and Population

This is a retrospective, case-control study utilizing baseline data from the Jordanian Atrial Fibrillation (Jo-Fib) registry that has the registration number of NCT03917992 [13]. The Jo-Fib study is a multi-centric, prospective, non-interventional, observational study registry. A total of 2000 patients aged ≥18 years were consecutively enrolled from 19 Jordanian hospitals, 30 out-patient cardiology clinics and one hospital in the Palestinian Territories. Patients’ enrollment started in May 2019 and concluded in November 2020. 

### 2.2. Measured Variables

Data were collected using standardized clinical forms relaying on personal interviews and medical charts. A team of researchers, including but not limited to consultants, specialists, residents, and medical students, extracted patients’ demographics, baseline clinical features, history of co-existing comorbidities, and medication history. The type of AF evident by 12-lead electrocardiography (EKG), Holter, or cardiac monitor was recorded for every patient upon enrollment. Participants were considered to have CAD if they have a documented acute coronary event, a history of typical/atypical cardiac chest pain with stress test findings, or coronary angiography findings suggestive of CAD. The collected data included patients’ demographics (age, gender, height (in cm), weight (in Kg), and BMI (kg/cm^2^)), symptoms of AF, co-morbidities (hypertension, diabetes, dyslipidemia, obstructive sleep apnea, heart diseases, stroke, chronic kidney disease, respiratory disease, thyroid disease, and cancer), history of systemic embolization, and medications history with emphasis on anticoagulants, antiplatelet agents, and antiarrhythmic medications. The researchers calculated the stroke risk using CHA2DS2-VASc score [14] and the bleeding risk using HAS BLED score for all patients [15]. Moreover, relevant Echocardiographic (ECHO) information (left ventricular ejection fraction (LVEF), left ventricular hypertrophy (LVH), left atrial size (in cm), presence of rheumatic heart disease, presence of pulmonary hypertension. and various other findings) were extracted. 

### 2.3. Data Analysis

All extracted data were entered on Excel sheets and further imported into STATA (Stata Statistical Software: Release 16. College Station, TX: StataCorp LLC 4905 Lakeway Drive College Station, TX 77845, USA). Differences in sociodemographic characteristics, cardiac medical history, and used medications between patients with history of CAD and patients with no history of CAD were assessed using chi-square test for categorical variables and Student t-test for continuous variables. Alternatively, nonparametric tests such as Kruskal–Wallis test were utilized to assess differences in continuous variables in the case of non-normally distributed data. A multistage binary logistic regression analysis was also used to assess for factors correlated with higher risk of CAD in atrial fibrillation patients. The estat vif command was used to exclude variables with high variable inflation factor (VIF) to minimize multicollinearity. A *p* value of ≤0.05 was set to be a significant difference.

### 2.4. Ethical Consideration

The ethical approval of this study was granted from the Medical Research Ethical Committee and the Institutional Review Board (IRB) at the University of Jordan (ethical approval code: 10/2021/415, approval date: 29 December 2020). All participants were informed about the nature of the study, and a written consent was obtained from all patients. The authors assert that all procedures contributing to this work comply with the ethical standards of the relevant national and institutional committees on human experimentation and with the Helsinki Declaration of 1975, as revised in 2008.

## 3. Results

### 3.1. Characteristics of the Sample

Of the 2000 consecutively enrolled patients, 11.35% had CAD. The baseline clinical features are shown in Table 1. A total of 64.47% of CAD-AF patients were males (*p* < 0.01), with the mean age of 68.7 years. A total of 22.21% of CAD-AF had a normal body mass index (BMI), 31.25% were overweight, and 31.25% were obese. Of the 227 AF patients with CAD, 82.38% had been diagnosed with hypertension (*p* < 0.01), 66.52% with hypercholesterolemia (*p* < 0.01), 56.83% with diabetes mellitus (*p* < 0.01), and 18.06% were cigarette smokers (*p* = 0.04).

### 3.2. Cardiac History and Pharmacotherapy

Of the 227 CAD-AF patients, 34.81% were shown to have paroxysmal AF, while 65.2% had non-paroxysmal AF (*p* = 0.6). A total of 94.28% of CAD-AF patients had non-valvular AF (*p* = 0.08). The cardiac history of AF patients is shown in Table 2. Palpitations (*p* < 0.01), fatigue (*p* < 0.01), dizziness (*p* = 0.03), and shortness of breath (*p* < 0.01) were more commonly found in non-CAD patients, while more CAD-AF patients experienced chest pain (*p* < 0.01) or were asymptomatic (*p* < 0.01). Heart failure (*p* < 0.01) and chronic kidney disease (*p* = 0.04) were more common in patients with CAD. However, left ventricular hypertrophy (*p* < 0.01) was observed more in non-CAD patients. CAD-AF patients had a lower left ventricular ejection fraction (*p* < 0.01), a higher CHA2DSVASc2 score (*p* < 0.01), and a higher HAS BLED score (*p* < 0.01).

Table 3 shows the pharmacotherapy used in our sample. Most of our sample (81.1%) were using anticoagulants whether alone (48.2%) or in combination with antiplatelet agents (33%). Only 13.6% of our population used an antiplatelet agent alone, while 5.2% did not use any sort of antiplatelet agents or anticoagulants. Patients with CAD-AF showed significantly higher use of antiplatelet agents and anticoagulants/antiplatelet agents combination compared to the rest of the sample (*p* < 0.01). Out of the 1625 patients who used anticoagulants, 61.1% used novel oral anticoagulants (NOACs) compared to vitamin K antagonists (38.9%). Patients with CAD had significantly higher use of NOACs (*p* = 0.02). Regarding the used antiplatelet agents, the most commonly used regimen was aspirin (67.2%) alone, clopidogrel (17.7%), and dual antiplatelet therapy (13.7%), respectively. Patients with CAD had significantly higher rates of clopidogrel and dual antiplatelet therapy use (*p* < 0.01).

### 3.3. Factors Associated with an Increased Risk of CAD in AF Patients

Table 4 shows factors associated with an increased risk of CAD in AF patients. Females had a lower risk of CAD (OR = 0.35, *p* < 0.01, 95% CI: 0.24–0.50). AF Patients with dyslipidemia (OR = 2.5, *p* < 0.01, 95% CI: 1.8–3.4), history of smoking (OR = 1.7, *p* = 0.02, 95% CI: 1.1–2.6), higher CHA2DS2-VASc score (OR = 1.5, *p* < 0.01, 95% CI: 1.4–1.7), low LVEF (OR = 0.98, *p* = 0.01, 95% CI: 0.97–0.99), congenital heart disease (OR = 6.4, *p* = 0.04, 95% CI: 1.09–37.9), or asymptomatic AF (OR = 1.9, *p* < 0.01, 95% CI: 1.3–2.6) had a higher risk for CAD. On the other hand, patients with history of stroke or systemic embolization (OR = 0.3, *p* < 0.01, 95% CI: 0.2–0.55), heart failure (OR = 0.57, *p* < 0.01, 95% CI: 0.36–0.89), or left ventricular hypertrophy (OR = 0.5, *p* < 0.01, 95% CI0.36–0.7) had lower risk for CAD development.

## 4. Discussion

Our study found that almost one out of every ten AF patient had coexisting CAD. The multivariable regression analysis showed that patients with history of smoking, HTN, DM, and dyslipidemia had higher risk for CAD. Moreover, AF patients with history of stroke/systemic embolization or heart failure had lower risk of CAD development. These unexpected findings could be explained be survival bias; most of those with AF and any of these comorbidities died earlier, and only patients with excellent control of cardiac risk factors and compliance with medications survived to participate in this study [16]. Regarding pharmacotherapy, patients with CAD-AF patients showed a higher use of anticoagulants/antiplatelet agents combination.

In our sample, approximately one in each ten AF patients had CAD. Although we reported a lower prevalence of CAD in AF compared to the literature, it is still much higher than the prevalence of CAD in the general population, which is estimated to be around 2% [2,7]. Some might argue that these lower numbers indicate favorable prognosis for AF or better control of cardiac risk factors in Jordan. These findings, however, might paradoxically be due to survival bias; CAD-AF patients die earlier because of poor risk-factors control [16]. Moreover, the co-presentation of CAD and AF could be deceiving, as CAD-AF patients in our sample were asymptomatic or presenting only with chest pain. Therefore, it is highly recommended to develop risk-estimation scores to stratify CAD risk in AF patients.

Our results showed that males had higher prevalence of CAD compared to females. It was reported that around 49% of males over 40 are at risk of CAD compared to 32% of females [17]. These differences could be attributed to biological factors, such as the cardiac protective role of female sex hormones. The increased risk of CAD in men could be explained, however, by the behavioral differences between males and females [18,19]. Studies have reported that males are more likely to delay their scheduled clinic visits; as a result, they present very lately. Overall, males have a lower perceived risk for illnesses, thus later health-seeking behaviors [18]. 

Chronic AF is associated with accelerated atherosclerosis due to the generalized inflammatory and pro-thrombotic status [12]. It is still unclear whether this systematic inflammation is due to AF solely or because of the higher prevalence of cardiovascular risk factors in AF patients [11]. Moreover, episodes of AF with rapid ventricular response might result in demand ischemia, predisposing CAD, and type 2 MI attacks [20]. According to our results, smoking, HTN, DM, and dyslipidemia were all significantly more prevalent in CAD-AF patients. This is consistent with most regional studies about CAD risk factors [21,22,23]. In a study conducted in Iraq on patients undergoing coronary angiography for CAD, being a male, smoking, hypertension, hyperlipidemia, and a family history of CAD were all linked with premature CAD [21]. Another similar study in Jordan found that DM and smoking were associated with CAD, although they did not find any significant association between HTN or dyslipidemia with CAD [22]. Alsaud et al. found that most patients with CAD in Jordan have a cluster of more than one cardiac risk factor [23]. Therefore, cardiac risk factors should be controlled aggressively in patients with AF, as they are linked to higher risk of CAD, MI, and, subsequently, might worsen long-term morbidity and mortality.

Based on the American Heart Association/American College of Cardiology/European Society of Cardiology (ACC/AHA/ESC), antithrombotic therapy is a class IA recommendation to prevent stroke in AF patients with moderate to high risk to thromboembolism [24]. According to our results, around 80% of AF patients were using oral anticoagulants, with around 60% using NOACs and 40% using vitamin K antagonists. It is anticipated that the use of NOACs will increase with time, given the wide therapeutic index and more stable pharmacokinetic profiles compared to vitamin K antagonists. Nevertheless, vitamin K antagonists are still used in many developed countries, owing to their cheap price and coverage by insurance companies. Moreover, Vitamin K antagonists are the first line agents in AF of valvular etiologies, including patients with mild to moderate mitral stenosis as well as patients with prosthetic valves [25]. 

On the other hand, almost half of our sample was using some sort of antiplatelet therapy, with aspirin being the most commonly used one. Patients with CAD had significantly higher rate of dual antiplatelet therapy (DAPT) use. According the current ACC/AHA/ESC guidelines, daily aspirin is recommended for all patients with documented history of CAD, as it has shown to significantly reduce the risk of future vascular events [26,27]. Other modifications to this regimen, in the forms of increasing the dose or adding other antiplatelet agents, might be utilized in patients with a cluster of other cardiovascular risk factors [28]. 

Interestingly, almost one-third of our sample were using the combination of anticoagulants and antiplatelet agents. Patients with CAD had significantly higher rates of using this combination. Although aspirin is not routinely recommended to be used in AF, it seems that many cardiologists in Jordan are aggressively anticoagulating AF patients, owing to the increased prevalence of cardiovascular risk factors in the Jordanian population [23]. Guidelines regarding antithrombotic treatment of CAD-AF are inconsistent, as they differ between North America and Europe [29]. For AF, oral anticoagulants are more effective compared to antiplatelet therapy in terms of stroke prevention [30]. However, antiplatelet agents (especially daily aspirin) are recommended in CAD [31]. In CAD-AF patients, both oral anticoagulants and antiplatelet agents should be ideally used. Unfortunately, this combination will increase the bleeding risk [32]. To aid in tailoring AF management, many scores and guidelines were proposed. The CHA2DS2-VASc is the most commonly used score to stratify AF patients in terms of future risks for stroke development [14]. Also, the HAS-BLED score is used to evaluate the risk of bleeding in patients being treated with anticoagulants for AF. Our results showed that patients with CAD had significantly higher CHA2DS2-VASc and HAS-BLED scores [15]. The double trouble of AF and CAD complications, including increased risk of thromboembolic events and bleeding in our sample, might result in a therapeutic dilemma for the treating physicians. Therefore, healthcare providers should follow a personalized approach with a multidisciplinary collaboration for each patient having these comorbidities. 

To the best of our knowledge, this is the largest cohort-based study in the region addressing the relationship between AF and CAD. Nevertheless, the major limitation of our study is the observational, retrospective pattern of data collection. This raises the concerns of recall bias and precludes temporal evaluation of risk factors and outcomes. However, the personal interviews were double-checked with medical charts in order to minimize this potential limitation. Moreover, evaluating the actual risk of bleeding and thrombosis requires long-term, longitudinal studies with specific mortality and morbidity end points. Another potential limitation might arise from sampling; our study was performed in specialized cardiology clinics and medical centers which might not reflect the actual situation of AF patients. Nevertheless, owing to the nature of the healthcare system in Jordan, where patients usually bypass primary healthcare providers and seek specialty clinics directly, we think that our results reflect the actual prevalence of CAD in AF patients. 

## 5. Conclusions

Our study showed that one in each ten AF patients had CAD. Smoking, HTN, DM, and dyslipidemia were all significantly more prevalent in patients with CAD-AF. Although they had a higher risk of bleeding (based on HAS-BLED score), patients with CAD-AF were using oral anticoagulants and antiplatelet agents more frequently. Physicians should evaluate the risk of CAD development in patients with AF, and cardiac risk factors should be controlled accordingly, as concurrent, untreated AF and CAD carry poor outcomes. We believe that this study adds valuable information regarding the prevalence, epidemiological characteristics, and pharmacotherapy of CAD-AF patients. Longitudinal, long-term, prospective studies are required to obtain a better understanding of the course of CAD in AF, hopefully aiding in formulating more specific guidelines and scores for pharmacotherapy in this population.

## Figures and Tables

**Table 1:** General demographic characteristics of 2000 patients with atrial fibrillation in terms of having coronary artery disease.

Variable	Category	*N* (%)	History of CAD (%)	No History of CAD (%)	*p* Value
N	Total	2000	227 (11.35)	1773 (88.65)	
Age		67.7 ± 13.2 *	68.7 ± 11.7	67.5 ±13.3	0.2
Sex					<0.01
	Male	933 (46.65)	147(64.76)	786 (44.33)	
	Female	1067 (53.35)	80 (35.24)	987 (55.67)	
BMI *					0.14
	<25	426 (23.4)	46 (22.1)	380 (23.5)	
	25–29	659 (36.1)	65 (31.3)	594 (36.8)	
	>30	739 (40.5)	97 (46.6)	642 (39.7)	
	Total	1824 (100)	208 (11.4)	1616 (88.6)	
Medical history					
	Hypertension	1486 (74.3)	187 (82.38)	1299 (73.26)	<0.01
	Diabetes mellitus	882 (44.1)	129 (56.83)	753 (42.47)	<0.01
	Hypercholesterolemia	878 (43.9)	151 (66.52)	727 (41.0)	<0.01
	Cigarette Smoking (Current)	273 (13.66)	41 (18.06)	232 (13.08)	0.04

* Not all patients had weight and height measurements.

**Table 2:** Cardiac history of 2000 patients with atrial fibrillation in terms of having coronary artery disease.

Variable	Category	*N* (%)	History of CAD (%)	No History of CAD (%)	*p* Value
Atrial Fibrillation Type					0.6
	Paroxysmal	727 (36.35)	79 (34.81)	648 (36.55)	
	Non-Paroxysmal	1273 (63.65)	148 (65.2)	1125 (63.45)	
Etiology of Atrial Fibrillation					0.08
	Valvular	177 (8.85)	13 (5.73)	164 (9.25)	
	Non-valvular	1823 (91.15)	214 (94.28)	1609 (90.75)	
Atrial Fibrillation Symptoms	Palpitations	859 (43)	74 (32.6)	785 (44.28)	<0.01
	Fatigue	420 (21)	32 (14.1)	388 (21.88)	<0.01
	Dizziness	226 (11.3)	16 (7.05)	210 (11.84)	0.03
	SOB	673 (33.65)	55 (24.23)	618 (34.86)	<0.01
	Syncope	45 (2.25)	3 (1.33)	42 (2.37)	0.31
	Chest pain	34 (1.7)	11 (4.85)	23 (1.3)	<0.01
	Asymptomatic	591 (29.5)	98 (43.18)	492 (27.75)	<0.01
Comorbid Diseases	Stroke or systemic embolization	313 (15.65)	34 (14.98)	279 (15.74)	0.77
	Heart failure	467 (23.35)	69 (30.4)	398 (22.45)	<0.01
	Left ventricular hypertrophy	707 (39.1)	60 (26.44)	647 (36.49)	<0.01
	Congenital heart disease	11 (0.55)	2 (0.89)	9 (0.51)	0.5
	Pulmonary hypertension	543 (27.2)	56 (24.67)	487 (27.47)	0.37
	Sleep apnea	76 (3.8)	6 (2.65)	70 (3.95)	0.3
	Chronic kidney disease	181 (9)	29 (12.78)	152 (8.57)	0.04
	Active malignancy	117 (5.85)	9 (3.97)	108 (6.09)	0.2
CHA2DS2-VASc score					<0.01
	Score 1 in women and 0 in men (1)	174 (8.7)	4 (1.77)	170 (9.59)	
	Score 2 in women and 1 in men (2)	251 (12.55)	15 (6.61)	236 (13.31)	
	Score ≥3 in women and ≥2 in men (3)	1575 (78.75)	208 (91.63)	1367 (77.1)	
HAS-BLED score					<0.01
	0	266 (13.3)	11 (4.85)	255 (14.38)	
	1	728 (36.4)	70 (30.84)	658 (37.11)	
	2	623 (31.15)	89 (39.21)	534 (30.12)	
	>3	383 (19.15)	57 (25.12)	326 (18.39)	
Echocardiographic findings					
	LVEF *	53.6 ± 12.4	49.4 ± 13.8	54.2 ± 12.1	<0.01
	Rheumatic mitral stenosis	177 (8.85)	13 (5.73)	164 (9.25)	0.08
	Metallic prosthetic valve	111 (5.55)	11 (4.85)	100 (5.6)	0.62

* Left Vetricular Ejection Fraction, presented as mean ± standard deviation.

**Table 3:** Pharmacotherapy of patients with atrial fibrillation in terms of having a history of CAD.

Variable	Category	*N* (%)	History of CAD (%)	No History of CAD (%)	*p* Value
Use of anticoagulants/antiplatelet					<0.01
	None	103 (5.2)	6 (2.7)	97 (5.5)	
	Anticoagulants alone	964 (48.2)	55 (24.2)	909 (51.3)	
	Antiplatelet alone	272 (13.6)	38 (16.7)	234 (13.2)	
	Anticoagulants+Antiplatelets	661 (33)	128 (56.4)	533 (30.1)	
The type of used anticoagulation					0.02
	Vitamin K Antagonists	632 (38.9)	56 (30.6)	576 (39.9)	
	NOACs	993 (61.1)	127 (55.95)	866 (60.6)	
	Total	1625 (100)	183 (11.3)	1442 (88.7)	
The type of used Antiplatelet agent					<0.01
	Aspirin	627 (67.2)	73 (44)	554 (72.2)	
	Clopidogrel	165 (17.7)	45 (27.1)	120 (15.7)	
	Other antiplatelet	13 (1.4)	2 (1.2)	11 (1.4)	
	Dual antiplatelet therapy *	128 (13.7)	46 (27.7)	82 (10.7)	
	Total	933 (100)	166 (17.8)	767 (82.2)	
Antiarrhythmics	Beta blockers	1603 (80.15)	191 (84.14)	1412 (79.64)	0.11
	Amiodarone	403 (20.15)	45 (19.82)	358 (20.19)	0.9
	CCB	199 (10)	12 (5.29)	187 (10.55)	0.01
	Digoxin	299 (15)	24 (10.57)	275 (15.51)	0.05
Others	RAAS	746 (37.3)	105 (46.26)	641 (36.15)	<0.01
	Diuretics	759 (38)	103 (45.37)	656 (37)	0.01
	Statins	727 (36.35)	151 (66.52)	576 (32.49)	<0.01

* It includes aspirin + another antiplatelet agent.

**Table 4:** Multivariate binary logistic regression analyses for factors associated with higher risk of CAD.

Variable	Subgroups	OR	*p* Value	95% Confidence Interval
Sex	Male (Ref)			
	Female	0.35	<0.01	0.24–0.50
Dyslipidemia		2.5	<0.01	1.8–3.4
Stroke or systemic embolization		0.3	<0.01	0.2–0.55
Heart failure		0.57	0.01	0.36–0.89
Left ventricular hypertrophy		0.5	<0.01	0.36–0.7
Congenital heart disease		6.4	0.04	1.09–37.9
Smoking		1.7	0.02	1.1–2.6
Asymptomatic		1.9	<0.01	1.3–2.6
CHADVASC score		1.5	<0.01	1.4–1.7
Left ventricular ejection fraction		0.98	0.01	0.97–0.99

## Data Availability

The data that support the findings of this study are available from The Jordan Atrial Fibrillation Study (Jo-Fib) but restrictions apply to the availability of these data, which were used under license for the current study, and so are not publicly available. Data are however available from the authors upon reasonable request and with permission of The Center for Strategic Studies.
